# Seismic Intensity Prediction with a Low-Computational-Cost Transformer-Based Tracking Method

**DOI:** 10.3390/s25206269

**Published:** 2025-10-10

**Authors:** Honglei Wang, Zhixuan Bai, Ruxue Bai, Liang Zhao, Mengsong Lin, Yamin Han

**Affiliations:** 1 Hebei Earthquake Agency, Shijiazhuang 050021, China; wanghl@hbdzj.gov.cn; 2 College of Information Engineering, Northwest A&F University, Xianyang 712100, China; baizhixuan2529@nwafu.edu.cn (Z.B.); bairx@nwafu.edu.cn (R.B.); zl011360@nwafu.edu.cn (L.Z.); menciuspaper@nwafu.edu.cn (M.L.)

**Keywords:** seismic intensity, low cost, transformer-based visual tracking, seismic station simulation

## Abstract

The prediction of seismic intensity in an accurate and timely manner is needed to provide scientific guidance for disaster relief. Traditional seismic intensity prediction methods rely on seismograph equipment, which is limited by slow response times and high equipment costs. In this study, we introduce a low-computational-cost transformer-based (LCCTV) visual tracking method to predict seismic intensity in surveillance videos. To this end, an earthquake video dataset is proposed. It is captured in the laboratory environment, where the seismic level is obtained through seismic station simulation. With the proposed dataset, a low-computational-cost transformer-based visual tracking method is first proposed to estimate the movement trajectory of the calibration board target in videos in real time. In order to further improve the recognition accuracy, we then utilize a Butterworth filter to smooth the generated movement trajectory so as to remove low-frequency interference signals. Finally, the seismic intensity is predicted based on the velocity and acceleration derived from the smoothed movement trajectory. Experimental results demonstrated that the LCCTV outperformed other state-of-the-art approaches. The findings confirm that the proposed LCCTV can serve as a low-cost, scalable solution for seismic intensity analysis.

## 1. Introduction

Seismic intensity serves as an indicator of the degree of damage to the ground and buildings when an earthquake occurs. Furthermore, it poses a threat to underground facilities, including critical infrastructures such as transportation tunnels [1,2] and even research facilities like the LHC and HL-LHC [3,4]. In this paper, we mainly study how to predict the seismic intensity based on the ground and buildings when an earthquake occurs. This plays a crucial role in assessing the disaster situation, formulating rescue plans, using earthquake early warning systems, etc. The early popular seismic intensity analysis methods [5,6,7,8] are based on underground signals (i.e., P waves or S waves) collected by sensors such as seismographs. Wu and Zhao [5] defined the magnitude of earthquakes based on the peak displacement ($P_{d}$) of the initial P wave in the first three seconds. Katakami and Iwata [7] proposed a real-time S-wave-detection method based on an improved short-term average/long-term average (STA/LTA) algorithm. Subsequently, machine learning algorithms [9,10,11] and deep learning algorithms [12,13,14] were utilized to effectively improve the accuracy of the magnitude prediction. For example, Abdalzaher et al. [11] introduced a machine learning (ML) strategy based on numerous linear and nonlinear models for the quick determination of earthquake intensity after 2 s from the P-wave onset. Mousavi et al. [13] proposed an attentive deep learning model for the simultaneous detection of earthquake signals and picking of the first P and S phases on single-station data recorded from the local epicentral distance. However, these approaches rely on point-source-based models, which estimate the warning area based on magnitude and distance rather than directly predicting the intensity. More sophisticated methods for seismic intensity prediction, such as visible ground-level vibrations, are predominantly in the research phase. Furthermore, they are costly and cannot be deployed on a large scale, resulting in the inability to quickly estimate earthquakes in some areas.

With the rapid development of computer vision technology and video surveillance networks, surveillance cameras are spreading throughout various regions, even villages. Researchers [15,16,17,18,19,20,21] have focused on estimating earthquake intensity through surveillance videos that recorded the occurrence of the earthquake. Huang and Yao [19] utilized the motion estimation method based on macro-block matching to obtain a motion vector sequence in earthquake videos. Then, the seismic intensity was predicted quickly by an empirical function. Yang et al. [21] firstly utilized image processing technologies to obtain the movement of different types of objects in images taken before and after an earthquake. The corresponding seismic intensity was finally obtained based on the conditions satisfied by the object movement information. Although the above methods have been proven to be feasible, these methods rely on traditional manual methods to extract object motion information, which limits their accuracy and applicability. Recently, some works [15,16,22,23] have explored damage assessment of post-earthquake buildings based on deep learning technologies. Jing et al. [23] proposed a YOLOv5s-ViT-BiFPN-based neural network to detect damaged rural houses due to destructive earthquakes in UAV images. Wen et al. [22] introduced a novel seismic damage rapid recognition method for structural and nonstructural components based on convolutional neural networks (CNNs). However, these damage rates cannot be directly correlated with seismic intensity. Seismic intensity prediction based on deep learning has rarely been studied.

In this paper, our contributions are threefold:

- A low-computational-cost transformer-based tracking method (LCCTV) is proposed to predict seismic intensity in surveillance videos quickly.
- An efficient module (SMSA, Skip Multi-head Self-Attention) is designed to skip the MSA (Multi-head Self-Attention) computation in layers with high similarity. This reduces redundant self-attention operations and further lowers the overall computational cost of the model.
- The earthquake video dataset captured in the laboratory environments is introduced for open benchmark verification. Experimental results on the collected earthquake video dataset have shown that the proposed LCCTV method achieved superior performance in comparison to other state-of-the-art methods. Furthermore, it can run on edge devices in real time, demonstrating its practicality and effectiveness for earthquake intensity prediction.

To better expound our contributions, we conducted a comparison with other Transformer-based trackers. AsymTrack [24] focuses on using different convolution operations to mimic attention mechanisms in order to balance speed and accuracy. Its core is the Efficient Template Modulation (ETM) module, which employs dynamic 1D convolution to approximate multi-head cross-attention. In contrast, LCCTV emphasizes lightweight attention operations. Without altering the attention mechanism itself, it skips redundant and highly similar computations to achieve both high speed and high accuracy. Its core components are the Skip Multi-head Self-Attention (SMSA) module and mixed cross-attention. Mixformer [25], built on a one-stream Transformer, introduces the Mixed Attention Module (MAM) with asymmetric attention to reduce cost. However, due to redundant template computation and one-stream limitations, it fails to reach real-time speed on edge devices. By employing a two-stream structure and computing template features only once, LCCTV eliminates redundancy and delivers both higher accuracy and significantly faster runtime than MixedFormer.

## 2. Data Description and Processing

### 2.1. Data Collection

**Figure 1:**
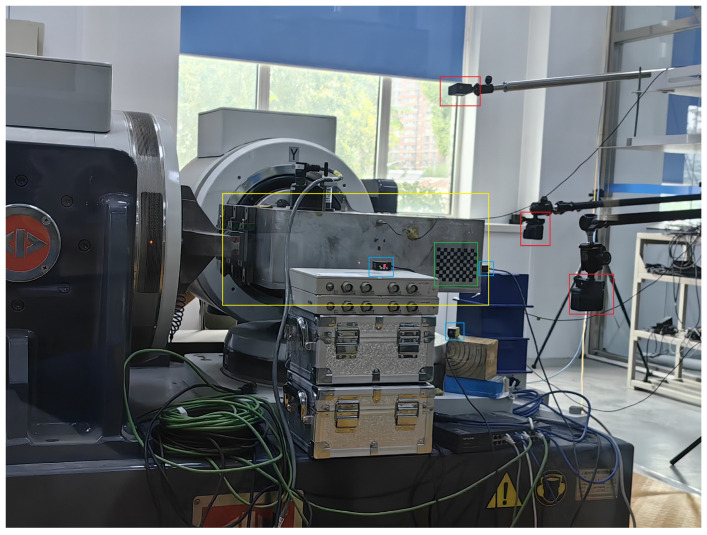
Data collection environment. The yellow box represents the earthquake simulation shaking table, the red boxes indicate the three cameras, the blue boxes mark the three laser displacement sensors, and the green box denotes the checkerboard calibration target.

The data used in this experiment were collected at the Institute of Engineering Mechanics, China Earthquake Administration. The data acquisition setup is shown in Figure 1. Centered around the earthquake simulation shaking table, three cameras and three laser displacement sensors were installed along the X, Y, and Z axes, respectively. The shaking table converts input digital signals into actual seismic waves to replicate earthquake scenarios. The cameras are used to record video footage of the seismic events generated by the shaking table, while the laser displacement sensors capture displacement data of the table. Additionally, three checkerboard calibration targets were affixed to the three platforms of the shaking table to serve as tracking targets. The earthquake simulation shaking table used in this study is model 3ES-20, with a platform size of 600 × 600 mm. It has a uniaxial sinusoidal rated excitation force of 20 kN and a maximum uniaxial random frequency of 1200 Hz. The maximum no-load performance in three directions includes an acceleration of 120 m/s^2^, a velocity of 1.2 m/s, and a displacement of ±19 mm. The three cameras used are GoPro models, and the laser displacement sensors are HG-C1100 units. Each checkerboard target measures 12 × 12 cm, with individual squares sized at 10 mm. The cameras were positioned 1 m away from the shaking table’s surface, directly facing the checkerboard targets, while the laser displacement sensors were placed 10 cm from the shaking table’s surface. Video data captured by the cameras were stored on memory cards, and the displacement data from the laser sensors were transmitted to a laptop via a data acquisition card.

A total of 84 sets of seismic data were collected, including 16 sets of sine sweep signals and 68 sets of real earthquake wave data. The real seismic data include records of earthquakes ranging from degrees II to VI, originating from locations such as Wenchuan, Wushi, and Maerkang.

### 2.2. Data Processing

The collected data included video (with a resolution of 1080p and a frame rate of 120 fps) footage captured by the GoPro cameras and voltage data from the laser displacement sensors, acquired through the data acquisition card. These two data types were processed separately. The video data was segmented according to the seismic platform’s designated vibration intervals, dividing long recordings into 60–80 s vibration clips. Each clip was labeled with the start time of the seismic platform’s operation. Due to the required warm-up period, the official start and end times of the simulated seismic waves were determined by adding the respective offset times to the platform’s start time. The segmentation of video clips is guided by the simulated start and end timestamps within each segment. Each video segment was further divided into individual frames, and the calibration targets within these frames were annotated using the labeling tool to build a seismic video dataset. Similarly, the voltage data collected by the data acquisition card was segmented according to the seismic platform’s vibration intervals.

Approximately 18 h of video data were processed, resulting in more than 700,000 images that cover the intensity of earthquakes from degree II to degree VI. The dataset included 3 sets of degree II earthquakes, 15 sets of degree iII, 26 sets of degree IV, 19 sets of degree V, and 20 sets of degree VI earthquakes.

## 3. Methods

### 3.1. Overview

**Figure 2:**
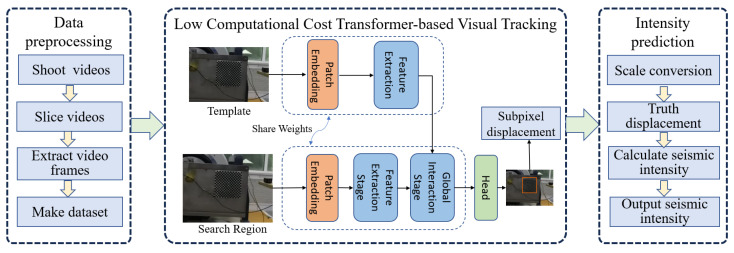
The overall framework. It consists of three modules: data processing, low-computational-cost transformer-based visual tracking, and intensity prediction.

An overview of the framework is shown in Figure 2. First, the collected video data is cropped and annotated to construct a tracking dataset, with the data collection and preprocessing procedures described in Section 2. Then, the LCCTV method, detailed in Section 3.2, is employed to efficiently extract vibration displacement. The resulting displacement is subsequently fed into the earthquake intensity prediction module, described in Section 3.2.1, which outputs the final intensity degree.

### 3.2. Low-Computational-Cost Transformer-Based (LCCTV) Visual Tracking

**Figure 3:**
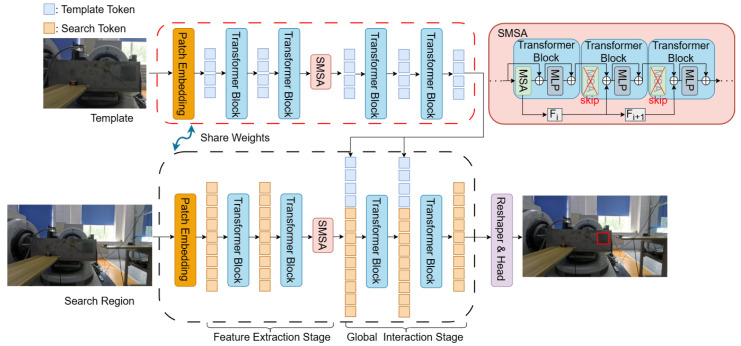
Overview of LCCTV. It employs an asymmetric Siamese pipeline, where the template branch runs once during initialization, generating the extracted template features of the last layer that are unidirectionally fed to the search region branch for global interaction.

As shown in Figure 3, LCCTV is a hybrid tracker that integrates both two-stream and one-stream architectures. It addresses the insufficient modeling of the relationship between the template and search region commonly found in dual-stream trackers, while also reducing redundant template computations typical of single-stream trackers, thereby significantly lowering computational cost. First, the template branch runs only once during initialization, and the extracted template features from the final layer are cached. The search branch is divided into two stages: the feature extraction stage and the global interaction stage. In the feature extraction stage, only the search region undergoes feature extraction without interacting with the template features, thereby reducing redundant template computations. In the global interaction stage, the cached template features are concatenated with the search features and fed into the transformer block for interaction. Meanwhile, we incorporate the SMSA module into both the template and search region feature extraction stages. By skipping MSAs with high similarity, this module reduces redundant self-attention computations and further lowers the overall computational cost. Finally, only the part that belongs to the search of the final sequence is selected, flattened into a 2D feature map, and fed into the head network for the tracking result.

#### 3.2.1. Feature Extraction

To avoid redundant template computations, we adopt an asymmetric two-stream architecture with shared weights, which separately extracts features from the template and the search region. For the given template $Z\in R^{3\times H_{z}\times W_{z}}$ and search region $X\in R^{3\times H_{x}\times W_{x}}$, the patch-embedding layer transforms them into a sequence of tokens $Z_{t}\in R^{C\times \frac{H_{z}}{16}\times \frac{W_{z}}{16}}$ and $X_{t}\in R^{C\times \frac{H_{x}}{16}\times \frac{W_{x}}{16}}$. During the feature extraction stage of the template and search region branch, the main computations in each Transformer encoder layer are

(1) $$Attn=softmax(\frac{QK^{T}}{\sqrt{d_{k}}})V$$

In the formula, for template tokens, $Q_{Z}=Z_{t}\cdot W_{Q}\left(W_{Q}\in R^{d_{k}\times \frac{H_{z}}{16}\times \frac{W_{z}}{16}}\right),K_{Z}=Z_{t}\cdot W_{K}\left(W_{K}\in R^{d_{k}\times \frac{H_{z}}{16}\times \frac{W_{z}}{16}}\right),V_{Z}=Z_{t}\cdot W_{V}\left(W_{V}\in R^{d_{v}\times \frac{H_{z}}{16}\times \frac{W_{z}}{16}}\right)$, where $d_{k}$ and $d_{v}$ are the dimensions of the projected key and value vectors.

In addition, we incorporate the SMSA module in this stage to reduce the computation cost. Inspired by [26,27], we observe that self-attention in adjacent layers of state-of-the-art language models exhibits very high similarity. Based on this observation, we represent MSA layers with high similarity using the MSA features from their neighboring layers, eliminating the need for repeated self-attention computation. This approach effectively skips the MSA computation, so we refer to it as SMSA. To ensure that using features from adjacent layers does not compromise the translation invariance and equivariance properties of the MSA block, we design a simple transformation function F. The function consists of an efficient channel attention module, two linear layers, and a depth-wise convolution in between them as follows:

(2) $$Feature_{i}^{MSA}=ECA\left(Linear_{2}\left(Conv\left(Linear_{1}\left(Feature_{i-1}^{MSA}\right)\right)\right)\right)$$

First, the MSA features from the previous layer $Feature_{i-1}^{MSA}$ are fed into the first linear layer $Linear_{1}:R^{n\times d}\to R^{n\times 2d}$ to expand the channel dimension. Then Conv is used with the kernel r×r to capture cross-token relations. The output is flattened back to a vector and fed into the last linear layer $Linear_{2}:R^{n\times 2d}\to R^{n\times d}$ to reduce the channel dimension back to the original size *d*. Finally, ECA is applied to enhance the cross-channel dependencies, producing the MSA features for the current layer $Feature_{i}^{MSA}$.

#### 3.2.2. Global Interaction

To enhance the modeling of relationships between the template and the search region, we introduce a global interaction stage in the search branch. In this stage, the last layer’s template features extracted from the template branch are concatenated with the search features obtained after the feature extraction stage in the search branch, and the combined features are used as the input to the encoder. Inspired by [25], we used mixed cross-attention to replace self-attention, where the query *Q* is generated solely from the search features. The mixed cross-attention can be expressed as:

(3) $$MixedCrossAttn=softmax\left(\frac{\left[Q_{x}\right]\left[K_{x}^{T};K_{z}^{T}\right]}{\sqrt{d_{k}}}\right)\left[V_{x};V_{z}\right]$$

In the formula, for the given template $Z\in R^{3\times H_{z}\times W_{z}}$ and search region $X\in R^{3\times H_{x}\times W_{x}}$, the patch embedding layer transform them into sequence of tokens $Z_{t}\in R^{C\times \frac{H_{z}}{16}\times \frac{W_{z}}{16}}$ and $X_{t}\in R^{C\times \frac{H_{x}}{16}\times \frac{W_{x}}{16}}$. $Q_{x}=X_{t}\cdot W_{Q}\left(W_{Q}\in R^{d_{k}\times \frac{H_{z}}{16}\times \frac{W_{z}}{16}}\right),K_{x}=X_{t}\cdot W_{K}\left(W_{K}\in R^{d_{k}\times \frac{H_{z}}{16}\times \frac{W_{z}}{16}}\right),K_{Z}=Z_{t}\cdot W_{K},V_{x}=X_{t}\cdot W_{V}\left(W_{V}\in R^{d_{v}\times \frac{H_{z}}{16}\times \frac{W_{z}}{16}}\right),V_{Z}=Z_{t}\cdot W_{V}$, where $d_{k}$ and $d_{v}$ are the dimensions of the projected key and value vectors.

#### 3.2.3. Head and Loss

We employ the center head [28] for prediction, which consists of three convolutional branches for center classification, offset regression, and size regression, respectively. The center classification branch outputs a centerness score map, where each score represents the confidence of the target center located at the corresponding position. The prediction of the offset regression branch is for the discretization error of the center. The size regression branch predicts the height and width of the target. The position with the highest confidence in the center score map is selected as the target position, and the corresponding regressed coordinates are used to compute a bounding box as the final prediction.

We adopt the weighted focal loss [29] for classification, and $l_{1}$ loss and the generalized IoU loss [30] are employed for bounding box regression. The overall loss function is

(4) $$L_{all}=L_{f}+\lambda_{iou}L_{iou}+\lambda_{l_{1}}L_{l_{1}}$$

where $\lambda_{iou}$ = 2 and $\lambda_{l_{1}}$ = 5 are regularization parameters following to balance optimization.

### 3.3. Earthquake Intensity Prediction

According to the China Seismic Intensity Scale [31], earthquake intensity prediction is based on the combined acceleration and combined velocity along three axes. This involves analyzing acceleration and velocity along three orthogonal axes—typically referred to as the x, y, and z axes—to accurately assess the overall motion characteristics of an earthquake event. First, we determine the scale between the pixel displacement and the real-world displacement using a checkerboard calibration target. For example, if the edge length of a square on the calibration target is 10 mm and it spans 24 pixels in the video, then 1 pixel corresponds to a real displacement of 0.417 mm. Based on this ratio, the pixel displacement obtained by LCCTV for the tracked target is converted into real-world displacement. With the frame rate of the video, we can determine the time interval between frames. Based on displacement and time, we calculate the velocity and acceleration of the target. We then compute the resultant (combined) velocity and acceleration. By integrating these components, we eventually estimate the intensity of the earthquake represented in the video. The detailed calculation process is as follows:

Calculate the combined triaxial acceleration records:

(5) $$a\left(t_{i}\right)=\sqrt{a^{2}{\left(t_{i}\right)}_{E-W}+a^{2}{\left(t_{i}\right)}_{N-S}+a^{2}{\left(t_{i}\right)}_{U-D}}$$

In the formula, $a(t_{i})$ represents the combined acceleration value at time point $t_{i}$, measured in meters per square second; $a{\left(t_{i}\right)}_{E-W}$ represents the filtered east–west directional acceleration value at time point $t_{i}$, measured in meters per square second; $a{\left(t_{i}\right)}_{N-S}$ represents the filtered north–south directional acceleration value at time point $t_{i}$, measured in meters per square second; and $a{\left(t_{i}\right)}_{U-D}$ represents the filtered vertical directional acceleration value at time point $t_{i}$, measured in meters per square second.

Calculate the combined triaxial velocity records:

(6) $$v\left(t_{i}\right)=\sqrt{v^{2}{\left(t_{i}\right)}_{E-W}+v^{2}{\left(t_{i}\right)}_{N-S}+v^{2}{\left(t_{i}\right)}_{U-D}}$$

In the formula, $v(t_{i})$ represents the combined velocity value at time point $t_{i}$, measured in meters per second; $v{\left(t_{i}\right)}_{E-W}$ represents the filtered east-west directional velocity value at time point $t_{i}$, measured in meters per second; $v{\left(t_{i}\right)}_{U-D}$ represents the filtered vertical directional velocity value at time point $t_{i}$, measured in meters per second; and $v{\left(t_{i}\right)}_{U-D}$ represents the filtered vertical directional velocity value at time point $t_{i}$, measured in meters per second.

Calculate the combined triaxial seismic parameters PGA (Peak Ground Acceleration) and PGV (Peak Ground Velocity). PGA represents the maximum value of the combined seismic acceleration records, measured in meters per second.

(7) $$PGA=\max[a\left(t_{i}\right)]$$

PGV represents the maximum value of the combined seismic velocity records, measured in meters per second.

(8) $$PGV=\max[v\left(t_{i}\right)]$$

The final intensity value is calculated based on PGA and PGV, with the formula as follows:

(9) $$I_{A}=3.17{log}_{10}\left(PGA\right)+6.59$$

(10) $$I_{V}=3.00{log}_{10}\left(PGV\right)+9.77$$

(11) $$I_{I}=\left\{\begin{array}{c} I_{V}\;I_{A}⩾6.0\;and\;I_{V}⩾6.0\\ \left(I_{A}+I_{V}\right)/2\;I_{A}<6.0\;or\;I_{V}<6.0 \end{array}\right.$$

$I_{A}$ denotes the calculated seismic intensity of using PGA. $I_{V}$ denotes the calculated seismic intensity of using PGV. $I_{I}$ denotes the final calculated seismic intensity.

## 4. Experiment

### 4.1. Experimental Environment

The experimental environment was based on Linux version 5.15.0-67-generic-amd64. The hardware included an Intel Xeon Silver 4316 CPU (Intel Corporation, Santa Clara, CA, USA), four NVIDIA GeForce RTX 4090 GPUs (24 GB each, NVIDIA Corporation, Santa Clara, CA, USA) and 128 GB (generic RAM) of system memory. The software environment was set up on Ubuntu 20.04.3 LTS, using Python 3.8 as the primary programming language, with PyTorch 1.13 and OpenCV as the main development libraries. The parallel computing environment was configured with CUDA 11.1.

### 4.2. Implementation Details

We use ViT-B [32] as our backbone and use the MAE [33] pretrained model to initialize it. We use four public datasets—GOT-10k [34], COCO [35], LaSOT [36], and TrackingNet [37]—as our training datasets. Image pairs are sampled from randomly selected video sequences, and common data augmentation techniques such as scaling and translation are applied to generate the pairs. We adopt the AdamW optimizer with a weight decay of $1\times 10^{-4}$. The initial learning rates for the backbone encoder and decoder are set to $4\times 10^{-5}$ and $4\times 10^{-4}$, respectively. The total number of training epochs is 300, and the learning rate is reduced to 10% of its original value after 240 epochs.

### 4.3. Algorithm Comparison

#### 4.3.1. Generic Tracking Benchmark

We benchmarked our proposed tracker, LCCTV, against several state-of-the-art (SOTA) methods on the LaSOT dataset [36], a large-scale and challenging benchmark for long-term visual object tracking. As summarized in Table 1, LCCTV achieves the highest performance among all compared methods, with 65.3% AUC, 75.4% P_*Norm*_, and 69.9% P. These results demonstrate the effectiveness of our approach in both robustness and accuracy under complex tracking scenarios. To further highlight our contributions, we conduct a detailed comparison with HiT-Base [38], a recent strong baseline based on hierarchical transformer design. LCCTV consistently outperforms HiT-Base by +0.7% in AUC, +2.1% in P, and +1.8% in P_*Norm*_, indicating better localization precision and tracking stability. In addition to accuracy, we also evaluate runtime efficiency across different hardware platforms. Specifically, we measure the inference speed of all trackers on a high-end Nvidia GeForce RTX 2080Ti GPU, which is manufactured by NVIDIA Corporation, Santa Clara, USA,”as well as on the Nvidia Jetson Orin NX 16 GB manufactured by NVIDIA Corporation, Santa Clara, USA, edge device, which is representative of practical deployment scenarios. As shown in Table 1, LCCTV achieves real-time performance on both platforms. Notably, on the Jetson Orin NX, our tracker maintains an average speed above 20 FPS, meeting the real-time performance standard, following the 20 fps real-time setting of VOT [39] challenge. This demonstrates the potential of LCCTV for resource-constrained real-world applications and validates the superiority of LCCTV in terms of both tracking accuracy and computational efficiency.

#### 4.3.2. Seismic Dataset Evaluation

In addition, we compared our seismic dataset (detailed in Section 2) with state-of-the-art trackers. According to the China Seismic Intensity Scale [31], earthquake intensity is classified into 12 degrees, represented by Roman numeral I to XII. In our earthquake dataset, the majority of the events fall within the intensity degrees II to VI. Specifically, there are 3 earthquake records with intensity degree II, 15 records with degree III, 26 records with degree IV, 19 records with degree V, and 20 records with degree VI. To assess the performance of different trackers in the context of seismic intensity prediction, we integrate each tracker with a downstream intensity estimation module, which maps the motion information extracted by the tracker to a predicted intensity degree. A prediction is deemed correct only if the predicted intensity degree exactly matches the ground-truth label. The prediction accuracy is then computed as the ratio of correctly predicted samples to the total number of samples.

The results are presented in Table 2. Our method, LCCTV, consistently achieves the highest prediction accuracy across all intensity degrees, significantly outperforming other state-of-the-art trackers. This indicates that LCCTV not only performs well on visual benchmarks but is also capable of capturing motion information that is critical for seismic assessment. Notably, as the intensity degree increases, the structural response becomes more complex and nonlinear. Despite this challenge, LCCTV maintains high prediction accuracy even at the degree VI, demonstrating its robustness and strong generalization to earthquake monitoring tasks. These results highlight the potential of LCCTV for deployment in earthquake monitoring systems, earthquake early warning systems, and post-earthquake damage assessment.

To verify the robustness and reliability of our method, in addition to the mean accuracy, we now also report the variance and the 95% confidence interval of the performance results, as shown in the Table 3. For example, at intensity degree VI, there were 20 test videos, among which 19 were correctly predicted, yielding an accuracy of 95.0%. The variance (0.047) and standard deviation (0.218) were calculated under the binomial distribution assumption, reflecting the dispersion of accuracy across samples. Furthermore, the 95% confidence interval (0.848–1.000) indicates that with 95% confidence, the true accuracy of our method for this intensity degree lies between 84.8% and 100%. These additional statistics provide a more rigorous quantification of performance.

#### 4.3.3. Visualization and Analysis of Earthquake Waveform Diagrams

To further validate the effectiveness and reliability of our method in earthquake intensity prediction, we conduct a detailed waveform-level analysis by visualizing the displacement–time curves generated during seismic events. These curves reflect the structural response over time and serve as a crucial indicator for assessing the accuracy of motion estimation in tracking-based seismic interpretation. We compare the predicted displacement–time waveforms obtained by LCCTV with two high-precision ground-truth references: The waveform generated by the earthquake simulation shaking table, which represents the input ground motion of the earthquake simulation shaking table, and the waveform measured by the laser displacement sensor, which captures the actual structural response.

**Figure 4:**
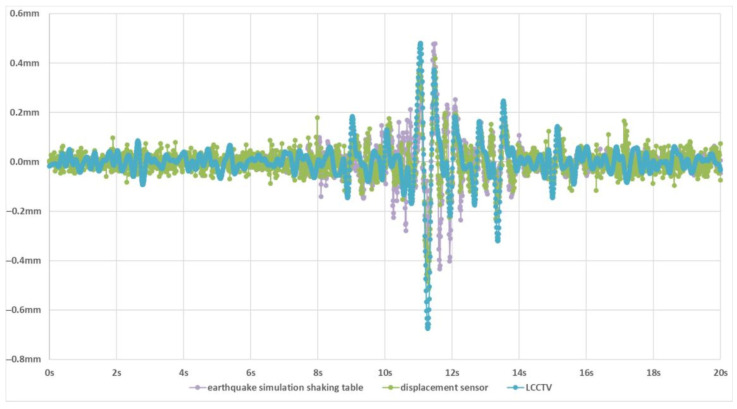
Displacement–time waveform. The earthquake simulation shaking table, displacement sensor, and LCCTV are represented by the purple, green, and blue lines, respectively.

**Figure 5:**
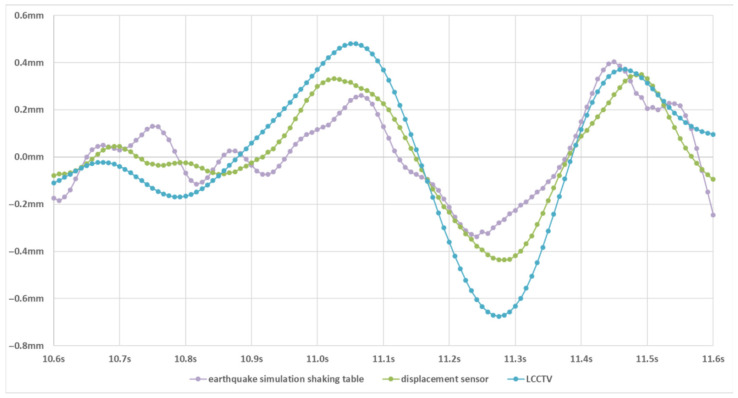
Waveform diagram at the peak. The earthquake simulation shaking table, displacement sensor, and LCCTV are represented by the purple, green, and blue lines, respectively.

As illustrated in Figure 4, the displacement–time curves predicted by LCCTV show remarkable agreement in both trend and amplitude with those from the shaking table and displacement sensor. The overall waveform morphology is consistent, including key oscillation patterns and the timing of major shifts. This consistency confirms that LCCTV can reliably track fine-grained structural displacements under dynamic seismic loads. To evaluate the precision of LCCTV around critical regions, we further visualize the waveform near the peak displacement, a key feature strongly correlated with the intensity degrees of the earthquake, as shown in Figure 5. In this zoomed-in view, LCCTV not only correctly captures the peak position and amplitude but also aligns well with the micro-fluctuations and phase dynamics observed in the reference signals. This level of alignment demonstrates that our method is capable of high temporal fidelity, which is essential for applications such as early warning, post-quake damage estimation, and real-time monitoring of critical infrastructure.

#### 4.3.4. Analysis of Real Earthquake Scenarios

**Figure 6:**
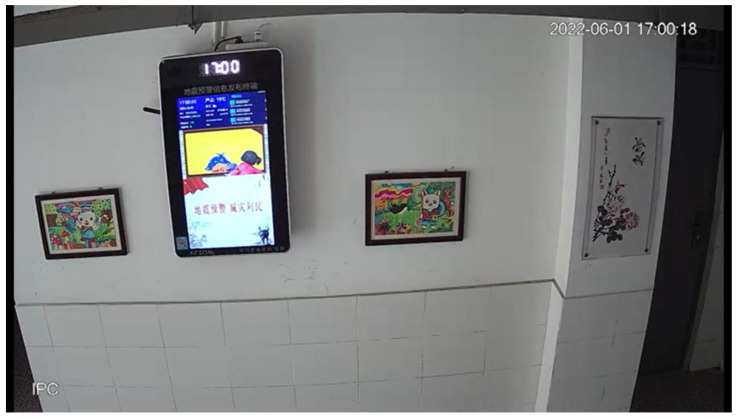
Real earthquake scenario in Lushan. The green box indicates the target tracked by our model.

**Figure 7:**
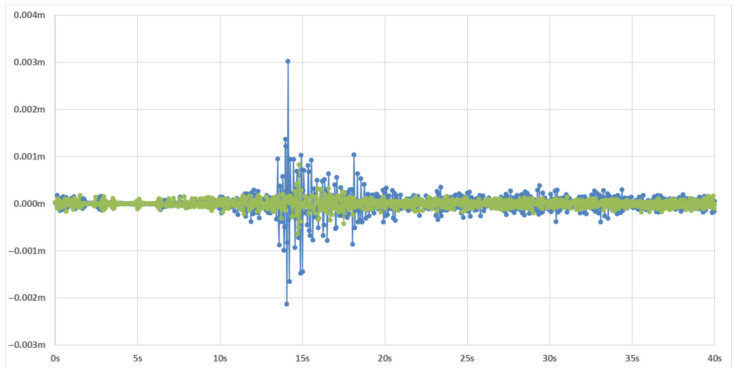
Displacement–time waveform diagrams in the X and Y directions. The green line represents the displacement in the X direction, and the blue line represents the displacement in the Y direction, respectively.

To further assess the practical applicability and robustness of our method, LCCTV, in real-world earthquake scenarios, we conducted an evaluation using actual seismic video footage from the magnitude 6.1 Lushan earthquake. This case study aims to verify whether LCCTV can accurately extract structural motion and predict seismic intensity outside controlled laboratory conditions. The selected video was captured in an indoor corridor environment, as illustrated in Figure 6, and reflects the kind of footage typically available from surveillance systems during seismic events. In this video, we focus on tracking the motion of a photo frame mounted on the wall, marked with a green bounding box. This object was chosen due to its clear visibility, fixed mounting, and high sensitivity to wall vibrations, making it an effective proxy for analyzing structural response. Using LCCTV, we extracted frame-by-frame displacement data of the tracked object to recover seismic motion in both the horizontal (X) and vertical (Y) directions. The resulting displacement–time waveforms are plotted in Figure 7. These waveforms exhibit clear and continuous oscillation patterns, with well-defined amplitudes and frequencies that correspond to typical ground-shaking behavior during moderate-to-strong earthquakes. Importantly, the estimated earthquake intensity derived from the displacement features captured by LCCTV is 6.0. The selected video was recorded at Luyang Town No. 2 Primary School in Lushan County, Ya’an City (latitude 30.147° N, longitude 102.9315° E), approximately 25 km south of the epicenter. According to the seismic intensity map of the M6.1 Lushan earthquake released by the China Earthquake Administration [47], this location falls within the region classified as intensity degree VI, which is consistent with the intensity estimated by LCCTV. This high level of agreement demonstrates that LCCTV is not only capable of tracking visual objects during intense motion but also possesses the precision and temporal resolution required to extract meaningful seismic parameters from video data alone.

**Figure 8:**
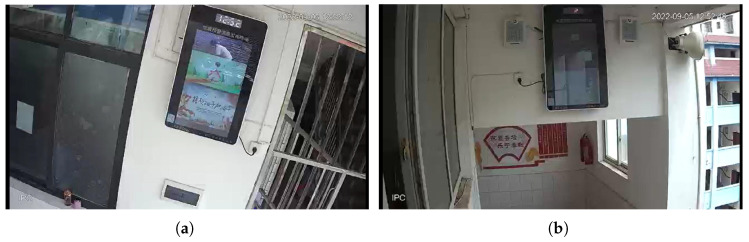
The two video scenarios of the Luding M6.8 earthquake. (**a**) Xinmian Town Primary School in Shimian County, Ya’an City (29.23° N, 102.35° E). (**b**) Ganluo Ethnic Primary School in Ganluo County, Liangshan Yi Autonomous Prefecture (28.97° N, 102.77° E).

To enhance the credibility of our method, we selected surveillance videos from two different locations with different intensity levels during the Luding M6.8 earthquake. The two videos were recorded at the Xinmian Town Primary School in Shimian County, Ya’an City (29.23° N, 102.35° E) in Figure 8a and the Ganluo Ethnic Primary School in Ganluo County, Liangshan Yi Autonomous Prefecture (28.97° N, 102.77° E) in Figure 8b. Using our LCCTV method, we calculated the earthquake intensity for both videos: 6.1 at Xinmian Town Primary School and 4.1 at Ganluo Ethnic Primary School. According to the instrumental intensity distribution measured by the Strong Motion Observation Group of the Institute of Engineering Mechanics, China Earthquake Administration, the instrumental intensity at Xinmian Town Primary School is 6.0, while that at Ganluo Ethnic Primary School is 4.2. These values are almost identical to those estimated by LCCTV, further demonstrating the robustness and accuracy of our method.

This experiment validates the generalization ability of LCCTV beyond a simulated environment and provides strong evidence of LCCTV’s capability to extract accurate motion signals directly from visual data, bridging the gap between vision-based tracking and traditional structural monitoring. Furthermore, it highlights the feasibility of using vision-based methods as an alternative or complement to traditional physical sensors, especially in environments where sensor installation is limited by cost, access, or preservation requirements. LCCTV offers a low-cost, high-accuracy alternative for seismic intensity analysis.

### 4.4. Ablation Study

To validate the effectiveness of the proposed SMSA module, we conduct detailed ablation studies by comparing the baseline model with its SMSA-integrated counterpart. The results are presented in Table 4. This ablation aims to assess whether SMSA can reduce computational complexity while preserving or enhancing the model’s tracking performance. As shown in the results, the integration of SMSA leads to a substantial reduction in both computational cost and model size: the number of MACs is reduced by 1.86 G, indicating a more efficient attention computation mechanism, and the number of parameters is reduced by 7.25 M, contributing to a more compact and deployable model. These results clearly demonstrate that SMSA serves as a lightweight yet effective replacement for traditional full self-attention, especially in transformer-based architectures where attention modules often dominate the overall complexity. By selectively skipping redundant attention operations, guided by high similarity between adjacent layers, SMSA enables the model to retain important long-range dependencies while eliminating computationally expensive and low-impact pathways. Importantly, this efficiency gain comes without compromising the overall performance of the tracker, as shown in the main results (refer to Table 1 and Table 2). This demonstrates that the proposed SMSA module strikes a favorable trade-off between model expressiveness and computational efficiency.

### 4.5. Failure Case and Limitations

Based on the results in Table 2, we observe that our method achieves strong overall performance but exhibits lower prediction accuracy for low-intensity earthquakes. In particular, we analyze misclassified cases for intensity degrees II and III, which are summarized in Table 5. The results show that degree II earthquakes were frequently misclassified as degree III, while degree III earthquakes were often misclassified as degree IV. To better understand these errors, we carefully reviewed the corresponding earthquake videos. For degree II events, the shaking was almost imperceptible, and the monitored objects showed negligible or no visible displacement. For degree III events, the shaking was slightly more evident but was still limited to minor oscillations with small amplitudes. Such subtle movements are difficult to capture reliably using our current tracking framework, as they often fall close to the noise threshold of video-based motion estimation. These observations suggest two key limitations of our current approach. One is sensitivity to micro-vibrations— the model struggles to detect and differentiate extremely small-scale object movements, which are critical for distinguishing between adjacent low-intensity degrees. The other is scale estimation inaccuracies—minor errors in object scale estimation accumulate when displacements are small, amplifying the risk of misclassification between adjacent intensity degrees. This highlights an inherent challenge in applying vision-based tracking for low-intensity seismic prediction, where the motion cues are weak and easily confounded by background noise, lighting variations, or camera stability.

**Figure 9:**
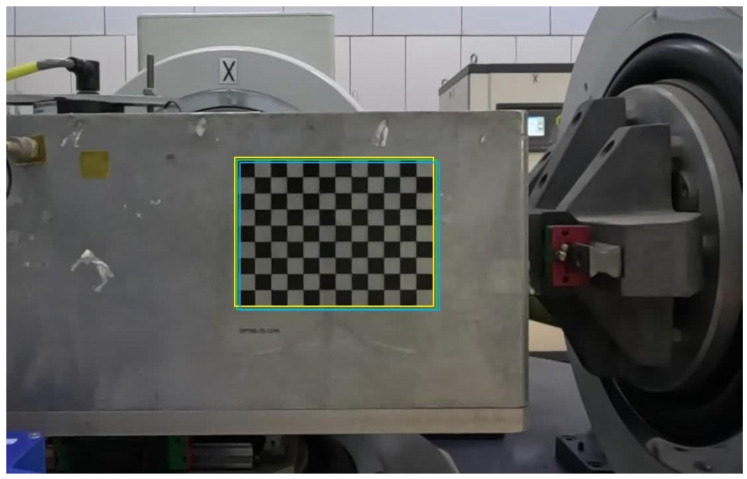
Tracking results of the video with intensity degree II. The green box represents the tracking result in the current frame, the yellow box corresponds to the next frame, and the blue box corresponds to the frame after that.

Specifically, we selected a video with an intensity degree of II for testing. As shown in Figure 9, the green box represents the tracking result in the current frame, the yellow box corresponds to the next frame, and the blue box to the frame after that. Under low-intensity conditions, object vibrations are negligible or show no visible displacement. However, as illustrated in Figure 9, due to noise and inaccuracies in scale estimation, the tracking boxes across the three frames exhibit noticeable displacements, resulting in errors. Since low-intensity cases are more sensitive to such errors, this ultimately leads to incorrect intensity predictions.

## 5. Conclusions

In this work, we present LCCTV, a pioneering approach to object tracking for seismic intensity prediction applications. LCCTV leverages a novel Siamese framework that combines the efficiency of two-stream designs with the performance benefits of one-stream architectures, while further introducing the lightweight SMSA module to reduce computational overhead. Our extensive experiments on both public tracking benchmarks and a dedicated seismic dataset validate the effectiveness of LCCTV. On the seismic dataset, LCCTV achieved an average prediction accuracy of 80.5%, significantly outperforming state-of-the-art trackers such as HiT-Base (72.2%) and AsymTrack-B (70.2%). In particular, our method demonstrated high robustness in medium- to high- intensity scenarios (e.g., 89.5% at degree V and 95.0% at degree VI), highlighting its ability to provide reliable predictions when structural vibrations become more pronounced. Moreover, LCCTV maintained real-time efficiency, which underscores its suitability for deployment on resource-constrained edge devices.

Despite its promising performance, LCCTV also exhibits certain limitations. Specifically, our method shows lower prediction accuracy at low-intensity earthquake degrees (II–III), where structural vibrations are extremely subtle and often difficult to distinguish from background noise. Moreover, the current framework still faces challenges in precise scale estimation under small displacements, which may lead to accumulated errors in intensity prediction. To address these issues, our future work will continue to build on the current foundation by incorporating sub-pixel motion estimation to enhance sensitivity to small vibrations and adopting more precise scale estimation to reduce the accumulation of prediction errors.

## Figures and Tables

**Table 1:** State-of-the-art comparison on LaSOT [36] dataset. The best three results are shown in red, blue and green fonts.

Methods	Source	LaSOT			Speed (fps)	
		AUC	P_*Norm*_	P	GPU	OrinNX
LCCTV	Ours	65.3	75.4	69.9	170	22
AsymTrack-B [24]	AAAI’25	64.7	73.0	67.8	197	41
MixFormerV2 [25]	NeurIPS’24	60.6	69.9	60.4	167	30
HiT-Base [38]	ICCV’23	64.6	73.3	68.1	175	39
E.T.Track [40]	WACV’23	59.1	-	-	53	21
HCAT [41]	ECCVW’22	59.3	68.7	61.0	205	34
FEAR [42]	ECCV’22	53.5	-	54.5	143	46
LightTrack [43]	CVPR’21	53.8	-	53.7	110	38
HiFT [44]	ICCV’21	45.1	52.7	42.1	213	50
ATOM [45]	CVPR’19	51.5	57.6	50.5	83	21
ECO [46]	CVPR’17	32.4	33.8	30.1	113	22

**Table 2:** State-of-the-art comparison with respect to our seismic dataset. The best three results are shown in red, blue and green fonts.

Tracker	II	III	IV	V	VI	Average
LCCTV (ours)	66.7	66.7	84.6	89.5	95.0	80.5
AsymTrack-B [24]	66.7	53.3	76.9	78.9	75.0	70.2
MixFormerV2 [25]	66.7	46.7	73.1	68.4	70.0	65.0
HiT-Base [38]	66.7	60.0	80.8	73.7	80.0	72.2
E.T.Track [40]	33.3	40.0	69.2	63.2	70.0	55.1
HCAT [41]	33.3	46.7	73.1	63.2	65.0	56.2
FEAR [42]	33.3	33.3	57.7	52.6	50.0	45.4
LightTrack [43]	33.3	40.0	61.5	57.9	55.0	49.6
HiFT [44]	0.0	26.7	46.2	42.1	50.0	33.0
ATOM [45]	33.3	33.3	50.0	47.4	45.0	41.8
ECO [46]	0.0	20.0	38.5	31.6	35.0	25.0

**Table 3:** The performance results of LCCTV.

Intensity Degree	Sample Size	Accuracy	Variance	Std. Dev.	95%S Confidence Interval
II	3	66.7	0.222	0.471	(0.000,1.000)
III	15	66.7	0.222	0.471	(0.406,0.928)
IV	26	84.6	0.130	0.361	(0.700,0.992)
V	19	89.5	0.094	0.307	(0.747,1.000)
VI	20	95.0	0.047	0.218	(0.848,1.000)

**Table 4:** Ablation studies of our LCCTV with or without SMSA.

	MACs (G)	Params (M)	Speed_GPU (fps)	LaSOT
w/ SMSA	14.15	54.88	170	65.3
w/o SMSA	16.01	62.13	152	64.6

**Table 5:** Failure Cases.

Truth	II	III	III	III	III	III
LCCTV (ours)	III	IV	IV	II	IV	IV

## Data Availability

The assessment data during this study is available in the Google Drive https://drive.google.com/drive/folders/1BZ5a40ZmIc0Y_eKCW6k_lVX_T3hDWCXZ?usp=sharing (accessed on 22 September 2025).
